# Comments on "Use of ChatGPT in Urology and its Relevance in Clinical Practice": Toward a Responsible AI Framework for Middle-Income Settings

**DOI:** 10.1590/S1677-5538.IBJU.2025.0459

**Published:** 2025-08-30

**Authors:** Juan Martín Montoya Osorio

**Affiliations:** 1 Pontificia Universidad Javeriana Bogotá Colombia Pontificia Universidad Javeriana, Bogotá, Colombia

To the editor,

We read with interest the article by Braga et al. evaluating ChatGPT's accuracy and reproducibility in pediatric urology (1). The study highlights important limitations: although ChatGPT can provide partially correct answers, it also produces incomplete, ambiguous, or potentially misleading responses—particularly regarding diagnostic definitions and treatment recommendations. We also noted a minor typographical error in the abstract ("those rwere evaluated qualitatively…"), which does not affect the results but may merit correction in the online version (1).

Another consideration is the rapid evolution of large language models (LLMs). The version evaluated by Braga et al. was most likely ChatGPT-3.5. Since then, GPT-5 has been released, offering improvements in contextual comprehension, medical reasoning, and response consistency. As a result, performance findings may change over short periods, underscoring the importance of continuous evaluation when clinical accuracy is critical.

These observations reinforce a broader point: deploying LLMs in clinical practice—particularly in middle-income countries—requires careful, evidence-based integration. In Colombia, the Interoperabilidad de la Historia Clínica Electrónica (IHCE), an Interoperable Electronic Health Record, provides an opportunity to structure and validate AI tools responsibly. Building on this context, we propose a five-point framework to guide the safe and effective adoption of AI in urology:

1. Prioritize validated, clinically relevant use cases—for example, prostate mpMRI interpretation and Gleason grading—where evidence is strongest (2, 3).
2. Enforce rigorous evaluation standards by adhering to TRIPOD+AI for predictive models and established AI trial reporting guidance (4).
3. Leverage national interoperability infrastructures (e.g., IHCE) to ensure data traceability, standardization, and privacy.
4. Adopt ethical safeguards recommended by the WHO for AI in health, including human oversight, transparency, and bias mitigation (5).
5. Implement short-term multicenter pilots measuring diagnostic accuracy, time to clinical decision, and AI-associated adverse events.

By learning from Braga et al.'s findings, addressing minor editorial issues, and accounting for rapid model evolution, urology in middle-income settings can advance from experimental enthusiasm to real-world, responsible integration. We believe these suggestions will resonate with IBJU's readership and provide a practical roadmap for countries facing similar challenges.

The Author

## Data Availability

Not applicable
